# Molecular Characterization of *Blastocystis* from Animals and Their Caregivers at the Gdańsk Zoo (Poland) and the Assessment of Zoonotic Transmission

**DOI:** 10.3390/biology10100984

**Published:** 2021-09-30

**Authors:** Monika Rudzińska, Beata Kowalewska, Małgorzata Waleron, Mirosław Kalicki, Katarzyna Sikorska, Beata Szostakowska

**Affiliations:** 1 Department of Tropical Medicine and Epidemiology, Faculty of Health Sciences, Medical University of Gdańsk, 80-210 Gdańsk, Poland; bkowal@gumed.edu.pl (B.K.); ksikorska@gumed.edu.pl (K.S.); 2 Laboratory of Plant Protection and Biotechnology, Intercollegiate Faculty of Biotechnology, University of Gdańsk and Medical University of Gdańsk, Abrahama 58, 80-307 Gdańsk, Poland; malgorzata.waleron@biotech.ug.edu.pl; 3 Zoological Garden of Gdańsk, Karwieńska 3, 80-328 Gdańsk, Poland; mkalicki@zoo.gda.pl; 4 Department of Tropical Parasitology, Faculty of Health Sciences, Medical University of Gdańsk, 80-210 Gdańsk, Poland; beata.szostakowska@gumed.edu.pl

**Keywords:** *Blastocystis*, subtypes, genetic diversity, molecular phylogeny, animals, humans, Poland

## Abstract

**Simple Summary:**

*Blastocystis* is one of the most common microorganisms living in the intestines of humans and various animals worldwide. Although the presence of this microorganism does not cause any ailments in many people, in some others *Blastocystis* is a source of various gastrointestinal disorders, such as abdominal pain, nausea, diarrhea, constipation, flatulence, or lack of appetite, as well as extraintestinal complaints, such as itching and skin rash. Transmission of *Blastocystis* is possible by direct contact with contaminated individuals, and by consuming water or food contaminated with cysts. It has been suggested that contact between animals and humans may pose a risk of human *Blastocystis* infection. In our study, we compared *Blastocystis* isolated from zoo animals and their keepers. The detection of identical sequences of *Blastocystis* in three monkeys and the man who looked after them showed that transmission of this microorganism between non-human primates (NHPs) and humans is possible under favorable conditions. Our research has shown that further investigation of animals and their in-contact humans is needed to better understand the transmission of *Blastocystis* between animals and humans and to find out which animals pose a risk of human infection, and to what extent.

**Abstract:**

*Blastocystis* is a highly genetically diverse gut protist commonly found in humans and various animals. The role of animals in human infection is only partly understood. The aim of this study was to determine the host specificity and possibility of zoonotic transmission of this microorganism. Subtypes of *Blastocystis* isolated from 201 zoo animals and their 35 caregivers were identified by sequencing of the SSU rRNA gene. *Blastocystis* was found in 26.86% of animal and 17.14% of human samples. Both mammalian (ST1–ST3, ST5, ST8, ST10, ST13, ST14) and non-mammalian subtypes were detected. Of the subtypes found in non-human primates (ST1, ST2, ST3, and ST13), two subtypes (ST1 and ST3) were also detected in humans. The presence of identical ST1 sequences in three monkeys and their caregiver indicates the possibility of direct transmission of *Blastocystis* between these animals and humans. Detection of ST5 only in wild boars and peccaries, ST8 only in Marsupial, ST10 and ST14 only in Bovidae, and non-mammalian subtypes in reptiles suggests higher host specificity for these subtypes, and indicates that their transmission between animals and humans is unlikely. Additionally, this was probably the first time that ST5 was found in peccaries, ST2 in patas monkeys, and ST8 in red kangaroos.

## 1. Introduction

*Blastocystis* is a cosmopolitan micro-eukaryote living in the intestines of humans and a wide range of animal species. *Blastocystis* probably infects over 1 billion people worldwide, and the reports on the influence of the organism on human health are contradictory [1]. On the one hand, human *Blastocystis* infection has been linked with the occurrence of intestinal complaints, such as nausea, abdominal pain, diarrhea, and flatulence; possible contribution of *Blastocystis* to the development of irritable bowel syndrome and/or cutaneous lesions has also been noted [2,3]. On the other hand, long-term asymptomatic *Blastocystis* carriage has been documented [4]. Others noted that colonization by *Blastocystis* was associated with the presence of more diverse and healthy gut microbiota than gut dysbiosis, hence *Blastocystis* carriage should not be viewed in isolation from the accompanying intestinal microbiome [5,6,7].

The mode of transmission of *Blastocystis* has not been fully elucidated; however, infection by the fecal–oral route via cyst-like forms, as well as by water and food contaminated with cysts, is considered the most probable means of infection [8].

Apart from humans, *Blastocystis* has been widely reported in various animal hosts including livestock, pets and wild animals, and animals living in zoos. Although *Blastocystis* isolates are indistinguishable morphologically, they show high genetic diversity. Based on variability within the small subunit of ribosomal RNA (SSU rRNA) genes, mammalian and avian *Blastocystis* isolates has been divided into 17–25 subtypes (STs) [9,10]. All of them (apart from ST9 sporadically found in humans) have been reported in different proportions in non-human primates (NHPs), other mammals, and birds [11,12,13,14,15,16,17]. Some subtypes found in animals with which humans come into frequent, but also less frequent, contact (e.g., ST5 most common in pigs, ST6 and ST7 in birds, and ST4 in rodents) are also reported in humans, while others (such as ST10 and ST14 common in cattle) are not found in humans [17,18,19]. The majority of human infections are caused by ST1–ST4, with a remarkable predominance of ST3, considered as a subtype of human origin. All this suggests diverse host specificity in *Blastocystis* subtypes and a possible zoonotic source for some human *Blastocystis* infections [20]. The evidence supporting the zoonotic potential of some *Blastocystis* subtypes includes the detection of very similar, or even identical, sequences of ST5 isolates in pigs or ST6 isolates in poultry, and in people who had contact with these animals [17,21], or the detection of ST2 isolates in both children and rhesus monkeys living in the same area [22]. Such a suspicion is also raised by the identification of ST8 in NHPs from a zoo along with their caregivers, or ST1, ST3, and ST4 in both pet animals and their owners [12,23]. The possibility of the human-to-animal transmission of *Blastocystis* has also been documented by successful attempts to infect rats, chickens, and gnotobiotic piglets with human *Blastocystis* isolates [24,25].

Further extensive molecular epidemiological research carried out on animals and their in-contact humans is needed to better understand the transmission of *Blastocystis* between animals and humans, and to find out which animals pose a risk of human infection, and to what extent.

In this study, we analyzed *Blastocystis* isolates from 201 animals representing 62 species kept in the zoological garden in Gdańsk (Poland), and from their caregivers, in order to better understand the host specificity of subtypes and the transmission of *Blastocystis* between animals and humans.

## 2. Materials and Methods

### 2.1. Sample Collection

Sampling was performed from November 2018 to April 2019 in the zoological garden located in northern Poland in Gdańsk, which covers an area of 125 hectares of landscaped park. The Gdańsk Zoo has almost 900 animals belonging to 164 species, and is visited by approximately 500,000 people a year. The animals are housed in large spaces in conditions as similar as possible to the natural habitat of each species. Some of the animals live alone in single cages, while others live in groups (for the map of locations of animals in the Gdańsk Zoo see this link: https://zoo.gda.pl/en/visit/zoo-map/) (assessed on 25 August 2021).

A total of 201 stool samples were gathered from different animals—mammals, birds and reptiles—as well as 35 stool samples from the humans who took care of them (Table 1). Prevalence values (percentage of animals infected), which are given with 95% confidence limits in parentheses (±CL_95_) were calculated by bespoke software “PERCENTAGE CONFIDENCE LIMITS VS 13” (courtesy of Dr. F.S. Gilbert and Prof. J.M. Behnke, University of Nottingham).

Animal stool samples (only fresh and after spontaneous defecation) were collected by their caregivers according to the zoo veterinarian’s guidelines during the daily morning cleaning of animals’ enclosures. Stool samples (placed into clean plastic containers) were transported to the laboratory a maximum of two hours later, and then stored in −20 °C until DNA extraction.

### 2.2. DNA Extraction and Amplification

Genomic DNA was extracted from animal and human stool samples, thawed immediately before extraction using the Genomic Mini AX Stool Kit (A&A Biotechnology, Gdynia, Poland) according to the manufacturer’s recommendation, and then stored at −20 °C until further processing.

The obtained DNA templates were examined for the presence of *Blastocystis* by amplification of an approximately ~620bp fragment of 18S rRNA gene (called barcode region) using the forward RD5 (5′-ATCTGGTTGATCCTGCCAGT-3′) and reverse BhRDr (5′-GAGCTTTTTAACTGCAACAACG-3′) primers [26] as described in [27].

### 2.3. Nucleotide Sequencing and Phylogenetic Analysis 

The PCR products were sequenced in both directions using a standard procedure with the primers used for amplification. The obtained sequences were assembled and aligned with the most similar *Blastocystis* sequences available at GenBank (in November 2020) using the MUSCLE algorithm with the default settings in Geneious Pro 9.1.8R (www.geneious.com, (assessed on 25 August 2021)). The alignment was edited manually to remove regions of ambiguity.

The phylogenetic analyses were performed with MEGA7 software (www.megasoftware.net, (assessed on 25 August 2021)) [28] using a maximum likelihood (ML) algorithm based on the Hasegawa-Kishino-Yano model [29] with 1000 bootstrap replicates. The best-fit model of nucleotide substitution was determined using the Akaike information criterion in Modeltest version 3.7 software [30]. The gene sequences of *Proteromonas lacertae* LA (NGBS01001136) were used as an outgroup. In order to classify the obtained sequences into STs, the open database for the classification of STs Blastocystis typing database (https://pubmlst.org/bigsdb?db=pubmlst_blastocystis_seqdef) (assessed on 25 August 2021) was used.

## 3. Results and Discussion

### 3.1. Infection Rate of Blastocystis

Of the 35 and 201 stool samples from humans and animals, respectively, six (17.14%) and 58 (28.43%) yielded PCR products congruent with *Blastocystis*. PCR products of *Blastocystis* isolates of four animals (two takins, a condor, and a rhea) were excluded from the phylogenetic analysis because of short length and poor quality despite the repetition of PCR, which ultimately yielded 54 (26.86%) animal representative sequences of *Blastocystis*. The percentage of infected animals in concerned host groups varied and was as follows: 80% in wild boars (Suidae), 90% in peccaries (Tayassuidae), 58.97% in NHPs, 34.78% in Bovidae, and 6.66% in Marsupials. Among reptiles, *Blastocystis* was detected in five of six turtles while in crocodiles, snakes and a *Heloderma* it was not found, resulting in a total of 41.66% of reptiles infected. No positive sample was found among carnivores and Aves (Table 1).

### 3.2. Detected Subtypes of Blastocystis

Among the six positive human samples, two subtypes, namely ST1 and ST3, were identified, each in three samples (50% each). In 49 out of 54 positive animal samples, eight subtypes were detected: ST1 (7.4%; 4/54;), ST2 (13%; 7/54), ST3 (11.1%; 6/54;), with ST13 (11.1%; 6/54) found only in NHPs, ST5 (31.5%; 17/54) only in wild boars and peccaries, ST10 (3.7%; 2/54) and ST14 (11.1%; 6/54) only in Bovidae, and ST8 (1.9%; 1/54) in a kangaroo. *Blastocystis* detected in five samples from turtles (9.3%) did not belong to any of the known mammalian and avian subtypes. The classification of the obtained sequences into STs based on the phylogenetic analysis was in agreement with the results of ST identification with the application of the open access bacterial population genomics: BIGSdb software, available on the PubMLST.org website. Detailed information on subtype distribution by the host is depicted in Table 2. Five of the eight subtypes detected in the zoo animals of Gdańsk (ST1, ST2, ST3, ST5, ST8; altogether 64.8%) are considered potentially zoonotic.

### 3.3. Genetic Diversity of Detected Blastocystis 

**Figure 1:**
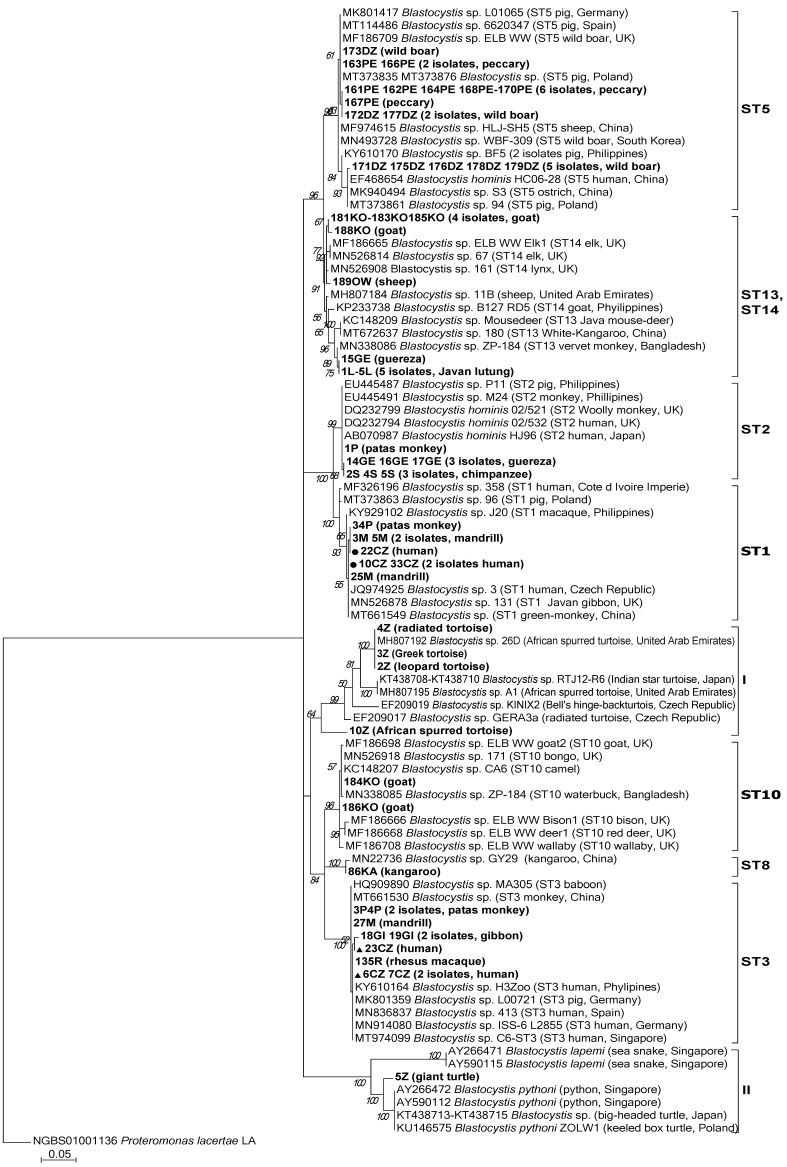
Molecular phylogenetic relationships of *Blastocystis* isolated from various animals and their caregivers from the Gdańsk Zoo. The phylogenetic analyses were performed with MEGA7 software using the maximum likelihood method and *Proteromonas lacertae* as an outgroup. Bootstrap values > 50% from 1000 replicates are shown on the nodes. The reference SSU rRNA *Blastocystis* sequences available at GenBank (in November 2020) are labeled with accession numbers, subtype number, the host, and locality if available. The *Blastocystis* sequences from this study with their host designations are shown in bold font. The sequences of Blastocystis ST1 and ST3 obtained from humans in this study are indicated by a dot and a triangle shape, respectively.

The results of phylogenetic analysis (Figure 1) showed that in the case of *Blastocystis* isolated from humans, only two subtypes, i.e., ST1 and ST3, were identified. *Blastocystis* of both of these subtypes found in humans were also detected in Old World monkeys, i.e., mandrills and patas (ST1) as well as in rhesus and gibbons (ST3).

**Figure 2:**
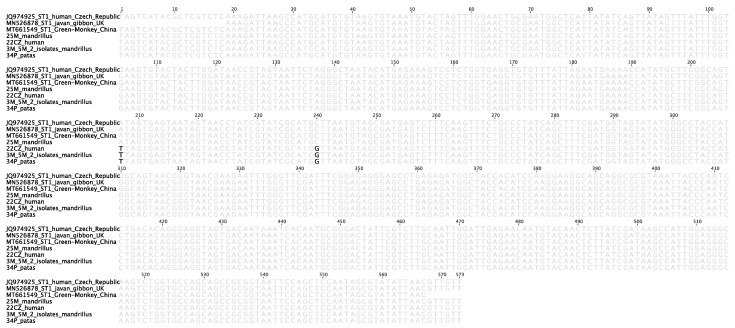
Sequence alignment of the 18S rRNA gene fragment of *Blastocystis* ST1 isolates. Nucleotides in bold represent SNPs at positions 207 and 240bp that are unique to Polish *Blastocystis* isolates 22CZ (from a human), 3M, 5M (from mandrills), and 34P (from a patas).

The ST1 sequence of a human (id. 22CZ) was identical with the sequences from two mandrills (id. 3M, 5M) and one patas (id. 34P). All of the four sequences had two single point mutations in the analyzed 574bp fragment of the 18S rRNA gene in comparison to *Blastocystis* sequences from GenBank, i.e., A-T transversion at the 207bp position and C-G transversion at 240bp, counting from the beginning of the alignment (Figure 2). The above-mentioned sequences are unique and have not been described hitherto in any other *Blastocystis* isolate apart from this study.

**Figure 3:**
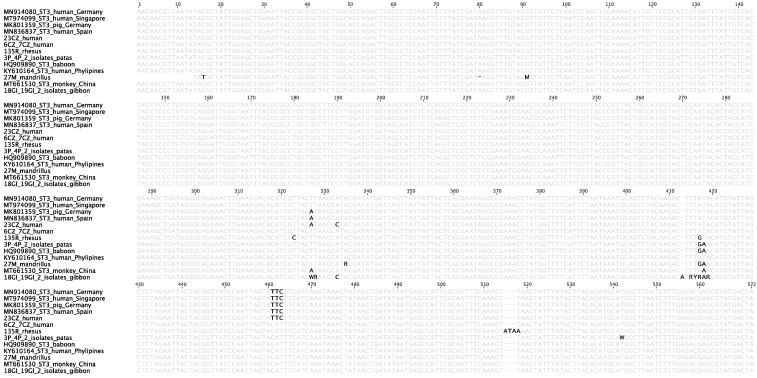
Sequence alignment of the 18S rRNA gene fragment of *Blastocystis* ST3 isolates. Nucleotides in bold represent unique mismatches with the reference sequences and insertions in the 18S rRNA gene sequence 135R of *Blastocystis* isolated from rhesus monkey.

The sequence analysis showed that the sequences of ST3 were variable. Among them, a total of 17 polymorphic positions were observed (Figure 3). Among ST3 sequences detected in zoo workers in Gdańsk (id. 6CZ, 7CZ, 23CZ), five different SNPs were observed. They were clustered together with other *Blastocystis* sequences originating from humans from different countries and with the sequences obtained in this study from mandrill (27M), rhesus (135R), gibbons (18GI, 19GI), and patas monkeys (3P and 4P). However, the characteristic insertion of three nucleotides ATA at the 515–517bp positions, counting from the beginning of alignment, was observed only in the *Blastocystis* isolate originated from rhesus (Figure 3). In addition, nine polymorphic positions were noted in the gibbons’ *Blastocystis* sequences, which may be the result of infection with different genetic variants of *Blastocystis*, as was reported by Vega et al. [31]. However, this hypothesis must be confirmed by cloning and separating different types of sequences.

Available data indicate that the infection rate of *Blastocystis* observed in both humans and animals varies depending on the study group, geographical region, and methods used in research. Since *Blastocystis* is a highly polymorphic organism and its cells are fragile and susceptible to damage, the use of molecular methods instead of microscopy provides more sensitive and accurate results, especially in low-intensity infections [1,32,33]. In the present study, the highest rate of *Blastocystis* infection, i.e., 80% and 90%, was observed respectively in wild boars and peccaries, followed by 58.97% in NHPs, and 34.78% in Bovidae (Table 1). Available reports on the occurrence of *Blastocystis* in wild boars recorded infection rates of 25% and 44% by microscopy [34,35] and 10.4%, 61.9%, and 76.9% when molecular methods were used [36,37,38]. A high infection rate for this protist (occasionally reaching 100%) has been described in the majority of reports concerning domestic pigs (closely related to wild boars) in various geographic regions of the world [21,27,39,40,41,42,43].

As for Bovidae, our study revealed *Blastocystis* in goats and sheep. In both cases, the observed infection rates of 87.5% (±CL_95_ 50.0–99.4) for goats and 50% (±CL_95_ 2.5–97.5) for sheep were in the upper range of *Blastocystis* prevalence previously recorded by different methods for these animals (0.3–94.7% for goats and 3.16–63.6% for sheep) [13,15,40,42,44,45,46,47,48,49]. The frequency of *Blastocystis* in NHPs in this study was in line with the data of many authors indicating that the percentage of infected individuals often exceeded 50% and even reached 100% [14,15,50,51,52,53].

As above-mentioned, ST5 was the only subtype detected in wild boars and peccaries in our study (Table 2). Similarly, ST5 was only observed in wild boars by Lee at al. [36], while Russini et al. [37], apart from ST5, recorded ST15 and a small number of ST3 in these animals. Hitherto ST5 was found mainly in pigs, and to a much lesser extent in other farm animals, such as cattle, goats, and sheep, which suggests that these animals are also sensitive to ST5 and may acquire this subtype from pigs when they live together or in close vicinity [11,19,45]. Occasionally, ST5 was also identified in humans working on pig farms with pigs harboring this subtype, indicating the possible transmission of ST5 from pigs to humans [21].

In our study, the sequences of all nine *Blastocystis* isolates obtained from peccaries and three isolates from wild boars were part of one clade. Along with these sequences, this clade included two sequences of *Blastocystis* isolates from pigs reared in Poland and from wild boars from Germany, Spain, and Great Britain. *Blastocystis* sequences from the remaining five wild boars from the Gdańsk Zoo clustered in the second clade along with *Blastocystis* sequences from a pig reared in Poland, and human and ostrich sequences from China (Figure 1). Of note, captive animals eat and defecate in the same relatively small space, so if one animal becomes infected, the rest of the animals in the herd can easily acquire the infection. This seemed to occur in the herds of wild boars and peccaries in our study, as the enclosures for peccaries and wild boars were adjacent to each other. Both the species enjoy foraging in the mud, which may have favored the spread of *Blastocystis* within and between herds. To our knowledge, only one study has reported the occurrence of *Blastocystis* in a peccary’s stool sample derived from the Center for the Conservation of Wild Fauna in Brazil. The sequence analysis confirmed the presence of *Blastocystis* in this sample; however, subtyping was unsuccessful [54]. Thus, our study was probably the first to identify the subtype of *Blastocystis*, namely ST5, in peccaries. It is worth noting that neither the people caring for wild boars and peccaries in the Gdańsk Zoo, nor other animals in the zoo, had ST5, which seems to confirm the high host specificity of this subtype. The obtained results confirmed that Suidae are the main hosts of ST5, and showed that peccaries, which belong to suborder Suina, like wild boars and pigs, are also susceptible to ST5.

ST10 and ST14 were detected in two and five goats tested, respectively. ST14 was also identified in one of the two sheep (Table 2). This is in line with other reports showing these subtypes as predominant in goats and sheep, and generally in wild and domesticated ruminants. Additionally, in previous studies, ST1, ST3–ST7, ST12, ST14, and ST15 were detected in these ruminants, but each of them much less frequently than ST10 and ST14 [13,19,44,45,46,47,49]. Importantly, goats and sheep from our survey stayed in the so-called “Little Zoo”—a separate enclosure where children can feed and touch tame animals, such as sheep, goats, and rabbits. Despite frequent contact with high numbers of zoo visitors, the goats and sheep did not acquire any subtype common in humans. Similarly, the results did not reveal any transfer of ST10 and ST14 from goats and sheep to their caregivers. This points out that ST10 and ST14 support a predilection for Bovidae while humans are not susceptible to these subtypes.

The sequence congruent to ST8 we identified only once—in one of the five red kangaroos sampled (Table 2). So far, a few kangaroos have been tested for *Blastocystis*, and different subtypes (ST4, ST10, ST12, ST13, and ST16) were identified in these animals [13,14,47,53,55]. To the best of our knowledge, this study was the first to identify ST8 in a red kangaroo. It follows that kangaroos are susceptible to infection with different subtypes and may constitute a reservoir of *Blastocystis*.

ST13 was detected in only two monkey species: in all five lutungs and in one of four guerezas (Table 2). Moreover, all six sequences were 100% identical (Figure 1). ST13 was observed in lutungs also by Li et al. [56] and in guerezas by Petrášová et al. [50]. Additionally, this subtype was also reported in golden snub-nosed monkeys [53] and vervet monkeys [50]. Originally ST13 was recognized in Australia in quokka (Marsupial) [55], followed by Western grey kangaroo [14], Java mouse-deer [15], and recently also in reindeer in China [48]. This shows that ST13, although rarely reported in animals, might have a wider host range. However, in the Gdańsk Zoo, this subtype was not detected in any of the animals, except for the mentioned species of monkeys. The presence of the identical sequences of ST13 in all tested lutungs and in only one of four guerezas (the remaining three had ST2) raises the question of whether the guereza acquired this subtype from the lutungs. Importantly, the enclosures of guerezas and lutungs in the Gdańsk Zoo are not adjacent to each other and are separated by the enclosures of patas and howler monkeys, in which ST13 did not occur. None of these species of monkeys had contact with each other in the zoo. Thus, it is highly likely that the ST13 was transferred from the yard of the lutungs to the yard of the guerezas by people caring for the monkeys, e.g., on their shoes.

Among the animals tested in this study, ST1, ST2, and ST3 were only detected in NHPs: ST1 in two species, ST2 in three species, and ST3 in four species (Table 2). To visualize the available data on *Blastocystis* STs in NHPs, we present the results obtained in monkeys from the Gdańsk Zoo and data reported for the same species by other authors in Table 3. These data show that among the eight subtypes reported hitherto in these species, ST1–ST3 clearly predominated, followed by ST5, while the remaining ST8, ST11, ST13, and ST15 occurred less frequently, or episodically.

It is worth noting that in our study in patas monkeys, three subtypes: ST1, ST2, and ST3 were identified, while in the only other study in which patas were tested, ST1 and ST3 alone were detected [57]. We found no other data on the occurrence of *Blastocystis* in patas, hence it appears that this study is the first to detect ST2 in patas monkeys. 

Of the eight *Blastocystis* subtypes identified in this study, only ST1 and ST3 were present in both humans and animals (specifically monkeys) while the remaining subtypes were only detected in animals. The presence of ST1 and ST3 in NHPs and humans suggests that these hosts are susceptible to infection with the same subtypes and therefore mutual contagion is possible. However, the mere presence of the same STs in animals and humans cannot be sufficient evidence for zoonotic transmission of *Blastocystis*. Stensvold et al. noted that the ST3 found in NHPs were more genetically diverse than the ST3 isolated from humans [62]. Evidently, in nature, as humans usually are not in contact with monkeys, the human and monkey *Blastocystis* gene pools do not mix and evolve separately. However, in one case of our study, we observed that not only the same subtype, namely ST1, but also the sequences of that subtype obtained from two mandrills and one patas, were identical to the sequence of ST1 isolated from the man who had contact with the monkeys (Figure 1). The highly probable transmission of *Blastocystis* from monkeys to humans was described by Stensvold et al. who identified ST8 (normally very rare in humans) in four out of sixteen animal handlers who had contacts with monkeys harboring this subtype [12]. In another study, the comparison of the 150bp variable region of the SSU rRNA gene of ST2 isolated from four children and rhesus monkeys living in the same area showed that the sequences were identical [22]. Our own and cited observations indicate that under favorable conditions, *Blastocystis* can spread between NHPs and humans, but it may be difficult or impossible to determine the direction of the transmission.

It is also worth noting that mandrills and patas monkeys (as is the case with lutungs and guerezas) have no contact with each other in the Gdańsk Zoo, and their enclosures are not adjacent to each other. This raises the suspicion that ST1 may have been transferred from the mandrill herd to the patas monkey by the humans caring for them, and confirms that related species are open to infection with the same subtypes. The relatively close relationship between monkeys and humans may explain the fact that the same subtypes (ST1, ST2, and ST3) were dominant in both of these hosts.

In this study, *Blastocystis* was not detected in any of the wild carnivores tested (Table 1). This is in concordance with several other reports [13,40,53,55,63]. In a few other studies this microorganism was found in wild carnivores, although the number of positive results in relation to the number of examined samples was always low, e.g., 4 of 213 Arctic foxes, 4 of 181 red foxes [64], 3 of 40 raccoon dogs [48], 1 of 7 African wild dogs [65], 1 of 4 grey wolves [15], 2 of 23 red pandas, and 10 of 81 giant pandas [66]. A similar low percentage of infected individuals were observed among wild felids, such as Scottish wildcats (1 of 13), lynxes (2 of 9) [16], white Bengal tigers (1 of 9), Siberian tigers (1 of 13) [65] and snow leopards (1 of 6) [14] as well as in carnivorous common genets (1 of 11) belonging to the Viverridae [64]. Our study included only single individuals of carnivores of different species, and this could have been the reason why *Blastocystis* was not detected in these animals. According to Farah Haziqah et al. [67], the factor responsible for the low frequency of *Blastocystis* in this group of animals may be the highly acidic pH in the gastrointestinal tract of carnivores (that may adversely affect the viability of *Blastocystis* cells). Although numerous subtypes of *Blastocystis* (ST1–ST6, ST8, ST10, ST14, and ST17) have been recorded in carnivores so far, none of them is specifically assigned to this group of animals. However, it is worth noting that in domestic dogs and cats, ST1–ST4 are predominant [68,69,70,71], which seems to suggest the possibility of *Blastocystis* transmission from humans to household dogs and cats. In the study of Nagel et al. [68] concerning people with gastrointestinal disorders and their dogs and cats, at least one common *Blastocystis* ST was observed in a pair: an animal and its owner.

As for birds, similar to carnivores, only single individuals of various bird species were tested in our study. Among them two samples (from a condor and a rhea) yielded products congruent with *Blastocystis*; however, their subtypes could not be determined due to unrecognized sequence data (Table 1). Until now, studies involved mainly chickens (*Gallus gallus domesticus*), while other domestic fowl and wild birds were examined less frequently. Among numerous subtypes recorded in birds (ST1, ST2, ST4–ST8, ST10, ST13, ST14, ST24, ST27, ST28), the vast majority were ST6 and ST7, considered therefore as “avian subtypes” [11,17,19,65,72,73,74]. Notably, the ST3, one of the most common subtypes in humans, has not been recorded in birds as yet, while ST6 and ST7, which are most common in birds, are rarely seen in humans [75,76]. Regarding wild and zoo birds, attention has been drawn by reports on the results of studies on ostriches, in which the ST5 was most often identified, but ST6, ST4 and ST10 were also observed sporadically [14,15,40,53,65,74]. In a study by Cian et al. [15] involving a group of over 70 birds of different families from two French zoos, *Blastocystis* was found in only seven samples. Similarly, from 109 samples of birds of different species, Maloney et al. found *Blastocystis* only in 16 of them [74]. This may suggest that *Blastocystis* in birds is either sparse or, for unknown reason, difficult to detect, and therefore may not have been detected in our group consisting of merely 25 individuals. To complete the picture of birds as a potential reservoir of *Blastocystis*, it should be added that the possibility of transmission of this microorganism between domestic birds (chickens, quails, and geese) as well as the infection of birds with human *Blastocystis* isolates has been documented [25,77].

Of the 12 reptile samples (each of a different species) tested in this study, *Blastocystis* was detected in five out of six turtles, resulting in 83.33% (±CL_95_ 41.1–99.1) of the infection rate in the Testudinate group (Table 1 and Table 2). Reptiles are a poorly studied group for *Blastocystis*. In the only report that included a larger group of turtles, i.e., 21 individuals, the percentage of infected animals was 28.5% [49]. Although the data on *Blastocystis* in reptiles are limited, they show a significant area of discrepancy between mammalian and avian *Blastocystis* sequences and those from reptiles, which may reflect evolutionary discrepancies between their respective hosts [15,49,78,79,80]. This discrepancy is also seen in our study in which *Blastocystis* isolated from turtles formed two independent clades, both distinct from those of mammals (Figure 1). The sequences of leopard, Greek, and radiated tortoises were identical and 99.49–99.66% concordant with the MH807192 sequence of a tortoise from the United Arab Emirates [49] with 7 SNPs. There is a high possibility of transmission of *Blastocystis* between these three tortoises in the Gdańsk Zoo, as they remain in full contact with each other during summer, when they are exposed together in the same outdoor enclosure and are fed with the same plant food (dandelion, iceberg lettuce, romaine lettuce, bananas, tomatoes, apples, beetroots, and parsley). Interestingly, the sequence of *Blastocystis* in the spurred tortoise, which also shares the enclosure with the above-mentioned tortoises in summer and eats the same food, differed significantly from their sequences and was only 85–91% concordant with other sequences of terrestrial herbivorous reptiles from GenBank (Figure 1). To explain the intriguing finding that the spurred tortoise maintained its own *Blastocystis* despite a similar lifestyle and sharing an enclosure with leopard, Greek, and radiated tortoises, further research on samples taken from turtles is needed. Another puzzling observation is that the sequence of *Blastocystis* in the giant Asian pond turtle was the only one that clustered separately together with the carnivorous aquatic reptile sequences: AY266472, AY590112 from pythons, KT438713-KT438715 from big-headed turtles, and KU146575 from keeled box turtle, with 100% bootstrap support (Figure 1). The giant turtle in the Gdańsk Zoo does not come into contact with other tortoises, and its diet, apart from above-mentioned plant food, includes beef. Under natural conditions, the diet of this turtle consists of worms, larvae, insects, snails, deceased animals, and aquatic and terrestrial plants [81]. It is highly probable that this omnivorous turtle kept in the Gdańsk Zoo hunts some of the invertebrates in its outdoor enclosure, which includes a pond, during the summer season. Therefore, it cannot be ruled out that this turtle became colonized by *Blastocystis* strains harbored by their victims. Hence, it would be worth examining the role of invertebrates in the transmission of *Blastocystis*. This supposition is supported by a recent study of cockroaches and golden monkeys living in the same area, in which in 82.8% of cockroaches tested, only the ST2 was found, while among three subtypes (ST1, ST2, ST3) detected in monkeys, ST2 was predominant [58].

## 4. Conclusions

Our study confirmed the occurrence of different *Blastocystis* STs in different hosts. The lower host specificity was observed in the cases of ST1 and ST3, which were detected in both humans and NHPs, showing that both humans and NHPs are susceptible to these subtypes. Additionally, the detection of the identical sequences of ST1 in three monkeys and the human who had contact with them demonstrated that under favorable conditions, direct transmission of *Blastocystis* between NHPs and humans is almost certainly possible. The detection of ST5 only in Suina, ST8 only in a marsupial, and ST10 and ST14 only in Bovidae indicates a higher host specificity for these subtypes, and a lower probability of infecting humans with them.

The shortcoming of our study is that for carnivores, birds, and reptiles, we had samples only from single animals of each species, which did not allow us to draw conclusions in relation to these groups of animals. Despite this, some interesting observations emerged from this study, making a valuable contribution to the full understanding of the circulation of *Blastocystis* between animals and humans, and the role of various animals as reservoirs for human infection. Future research should be expanded to free and captive reptiles (derived from zoos, reptile breeders, and reptile hobbyists) to improve the understanding of the genetic diversity, host specificity, and transmission patterns of *Blastocystis* in this poorly studied group of animals. Furthermore, in the search for sources of *Blastocystis* infection, research of invertebrates should be considered, as they may be a potential reservoir and/or a vector of *Blastocystis* infection for animals and humans. 

## Figures and Tables

**Table 1:** The total number of human and animal stool samples collected for this study and the percentage of positive samples obtained by the PCR method.

Host Name	Scientific Name	No. Examined	No. of Positives	% Positives (±CL_95_)
**Human**	*Homo sapiens*	**35**	**6**	**17.14 (8.2–31.3)**
**NHPs total**		**39**	**23**	**58.97 (43.0–73.7)**
Rhesus macaque	*Macaca mulatta*	1	1	
Bornean orangutan	*Pongo pygmaeus*	2	0	
Black howler	*Alouatta caraya*	4	0	
Chimpanzee	*Pan troglodytes*	5	3	
Patas monkey	*Erythrocebus patas*	5	4	
Mandrill	*Mandrillus sphinx*	8	4	
Javan lutung	*Trachypithecus auratus*	5	5	
Guereza	*Colobus guereza kikuyuensis*	4	4	
Buff-cheeked gibbon	*Nomascus gabriellae*	1	1	
White-cheeked gibbon	*Nomascus leucogenys*	1	1	
Emperor tamarin	*Saguinus imperator subgrisescens*	3	0	
**Carnivora total**		**20**	**0**	
**Canidae**				
European grey wolf	*Canis lupus*	2	0	
Maned wolf	*Chrysocyon brachyurus*	2	0	
Fennec fox	*Vulpes zerda*	1	0	
**Ursidae**				
Brown bear	*Ursus arctos*	1	0	
**Felidae**				
African lion	*Panthera leo bleyenberghi*	6	0	
Amur tiger	*Panthera tigris altaica*	2	0	
Persian leopard	*Panthera pardus saxicolor*	2	0	
Serval	*Leptailurus serval*	2	0	
**Procyonidae**				
Brown-nosed coati	*Nasua nasua*	2	0	
**Artiodactyla total**		**77**	**25**	**32.46 (22.1–44.6)**
**Suidae**				
Wild boar	*Sus scrofa*	10	8	
**Tayassuidae**				
Collared peccary	*Pecari tajacu*	10	9	
**Hippopotamidae**				
Pigmy hippopotamus	*Choeropsis liberiensis*	2	0	
Hippopotamus	*Hippopotamus amphibius*	1	0	
**Bovidae**		**23**	**8**	**34.78 (17.8–56.7)**
European wisent	*Bison bonasus bonasus*	5	0	
Yak	*Bos grunniens*	5	0	
Mishmi takin	*Budorcas taxicolor*	3	0 *	
Domestic goat	*Capra hircus*	8	7	
Polish heath sheep	*Ovis aries polish_heath*	2	1	
**Camelidae total**		**29**	**0**	
Alpaca	*Vicugna pacos*	8	0	
Llama	*Lama glama*	12	0	
Dromedary	*Camelus dromedarius*	5	0	
Bactrian camel	*Camelus bactrianus*	4	0	
**Perissodactyla**				
South American tapir	*Tapirus terrestris*	2	0	
**Metatheria total**		**15**	**1**	**6.66 (0,4–30.2)**
Red-necked wallaby	*Macropus rufogriseus*	10	0	
Red kangaroo	*Macropus rufus*	5	1	
**Leporidae** European rabbit **Rodentia**	*Oryctolagus cuniculus*	3	0	
Capybara	*Hydrochoerus hydrochaeris*	6	0	
Common gundi	*Ctenodactylus gundi*	1	0	
**Aves total**		**25**	**0**	
Eurasian eagle owl	*Bubo bubo*	1	0	
Great hornet owl	*Bubo virginianus*	1	0	
Greater rhea	*Rhea Americana*	5	0 *	
Violet turaco	*Musophaga violacea*	1	0	
Salmon-crested cockatoo	*Cacatua moluccensis*	1	0	
Military macaw	*Ara militaris mexicana*	2	0	
Yellow-crowned amazon	*Amazona ochrocephala ochrocephala*	1	0	
Greater flamingo	*Phoenicopterus roseus*	5	0	
Great curassow	*Crax rubra rubra*	2	0	
Southern ground hornbill	*Bucorvus leadbeateri*	3	0	
Andean condor	*Vultur gryphus*	3	0 *	
**Reptilia total**		**12**	**5**	**41.66 (18.1–70.6)**
Nile crocodile	*Crocodylus niloticus*	1	0	
Reticulate Gila monster	*Heloderma suspectum suspectum*	1	0	
African rock python	*Python sebae*	1	0	
Cuban tree boa	*Chilabothrus angulifer*	1	0	
Boa constrictor	*Boa constrictor*	1	0	
Yellow anaconda	*Eunectes notaeus*	1	0	
Leopard tortoise	*Stigmochelys pardalis*	1	1	
Spur-thighed tortoise (Greek tortoise)	*Testudo graeca*	1	1	
Giant Asian pond turtle	*Heosemys grandis*	1	1	
Radiated tortoise	*Astrochelys radiata*	1	1	
African spurred tortoise	*Centrochelys sulcata*	1	1	
Malaysian giant pond turtle	*Orlitia borneensis*	1	0	
**Animals total**		**201**	**54**	**26.86 (23.1–31.0)**

* excluded from phylogenetic analysis due to unresolved sequence data despite repeating PCR.

**Table 2:** Subtypes of *Blastocystis* detected in individual hosts. The ST are numbered according to ST designation in the PubMLST.org.

Sample id	Host	No of Identified Sequences	ST1 (n)	ST2 (n)	ST3 (n)	ST5 (n)	ST8 (n)	ST10 (n)	ST13 (n)	ST14 (n)	NMA ST (n)
6CZ, 7CZ, 10CZ, 22CZ, 23CZ, 33CZ	Human	6	3	-	3	-	-	-	-	-	-
	**NHPs total**	**23**	**4**	**7**	**6**	**-**	**-**	**-**	**6**	**-**	**-**
135R	Rhesus macaque	1	-	-	1	-	-	-	-	-	-
2S, 4S, 5S	Chimpanzee	3	-	3	-	-	-	-	-	-	-
1P, 3P, 4P, 34P	Patas monkey	4	1	1	2	-	-	-	-	-	-
3M, 5M, 25M, 27M	Mandrill	4	3	-	1	-	-	-	-	-	-
1L-5L	Javan lutung	5	-	-	-	-	-	-	5	-	-
14GE-17GE	Guereza	4	-	3	-	-	-	-	1	-	-
18GI	Buff-cheeked gibbon	1	-	-	1	-	-	-	-	-	-
19GI	White-cheeked gibbon	1	-	-	1	-	-	-	-	-	-
	**Suidae**										
171DZ-173DZ 175DZ-179DZ	Wild boar	8	-	-	-	8	-	-	-	-	-
	**Tayassuidae**										
161PE-164PE 166PE-170PE	Collared peccary	9	-	-	-	9	-	-	-	-	-
	**Bovidae total**	**8**	**-**	**-**	**-**	**-**	**-**	**2**	**-**	**6**	**-**
181-186KO 188KO	Domestic goat	7	-	-	-	-	-	2	-	5	-
189OW	Polish heath sheep	1	-	-	-	-	-	-	-	1	-
	**Metatheria**										
86KA	Red kangaroo	1	-	-	-	-	1	-	-	-	-
	**Reptilia total**	**5**	**-**	**-**	**-**	**-**	**-**	**-**	**-**	**-**	**5**
2Z	Leopard tortoise	1	-	-	-	-	-	-	-	-	1
3Z	Spur-thighed tortoise (Greek tortoise)	1	-	-	-	-	-	-	-	-	1
5Z	Giant Asian pond turtle	1	-	-	-	-	-	-	-	-	1
4Z	Radiated tortoise	1	-	-	-	-	-	-	-	-	1
10Z	African spurred tortoise	1	-	-	-	-	-	-	-	-	1
	**Animals total**	**54**	**4**	**7**	**6**	**17**	**1**	**2**	**6**	**6**	**5**

**Table 3:** Comparison of *Blastocystis* subtypes identified in NHPs in this study with data obtained by other authors; A—monkeys native to Africa, As—monkeys native to Asia.

Host	*Blastocystis* STs								Reference
Chimpanzee (A) *Pan troglodytes*	**-**	**ST2**	**-**	**-**	**-**	**-**	**-** - - - - - - - - - -	**-**	**This study**
	- - - ST1 - ST1 ST1 ST1 ST1 ST1	ST2 ST2 ST2 - ST2 - - - - ST2	ST3 - - - - - - - - ST3	ST5 ST5 - - - ST5 - - - ST5	- - - - - - - - - -	- - - ST11 - - - - - -		ST15 - - - - - - - - -	[57] [15] [38] [14] [53] [58] [59] [11] [50] [12]
Buff-cheecked gibbon (As) *Nomascus gabriellae*	**-**	**-**	**ST3**	**-**	**-**	**-**	**-** - - - - -	**-**	**This study**
	ST1 ST1 ST1 ST1 ST1	- ST2 - - -	ST3 St3 - - ST3	- ST5 - -	- ST8 - -	- - - - -		- ST15 - - -	[57] [16] [15] [58] [12]
White-cheecked gibbon (As) *Nomascus leucogenys*	**-** - ST1	**-** ST2 -	**ST3** ST3 -	**-** - -	**-** - -	**-** - -	**-** - -	**-** - -	**This study** [60] [57]
Mandrill (A) *Mandrillus sphinx*	**ST1**	**-**	**ST3**	**-**	**-**	**-**	**-** - - - - -	**-**	**This study**
	ST1 ST1 - ST1 -	- - - - -	- - ST3 ST3 ST3	- - - - -	- - - - -	- - - - -		- - - - -	[57] [15] [53] [58] [54]
Rhesus macaque (As) *Macaca mulatta*	**-**	**-**	**ST3**	**-**	**-**	**-**	**-** - - - - - - - -	**-**	**This study**
	ST1 ST1 ST1 ST1 - - - ST1	ST2 - ST2 ST2 ST2 ST2 - ST2	ST3 - ST3 ST3 ST3 ST3 - -	- - - - - - ST5 -	- - - - ST8 - - -	- - - - - - - -		- - - - - - - -	[53] [60] [61] [56] [38] [58] [11] [22]
Guereza (A) *Colobus guereza*	**-**	**ST2**	**-**	**-**	**-**	**-**	**ST13** - - ST13	**-**	**This study**
	- ST1 ST1	- ST2 ST2	ST3 - ST3	- - -	- - -	- - -		- - -	[57] [15] [50]
Patas monkey (A) *Erythrocebus patas*	**ST1**	**ST2**	**ST3**	**-**	**-**	**-**	**-** -	**-**	**This study**
	ST1	-	ST3	-	-	-		-	[57]
Javan lutung (As) *Trachypithecus auratus*	**-**	**-**	**-**	**-**	**-**	**-**	**ST13** - ST13	**-**	**This study**
	- ST1	- -	ST3 -	ST5 -	- -	- -		- -	[57] [56]

## Data Availability

The sequences obtained and analyzed during this study were deposited in the GenBank database under the accession numbers MW682185–MW682218.
